# Potent Attractant for Root-Knot Nematodes in Exudates from Seedling Root Tips of Two Host Species

**DOI:** 10.1038/s41598-018-29165-4

**Published:** 2018-07-18

**Authors:** Rasa Čepulytė, Wiseborn B. Danquah, George Bruening, Valerie M. Williamson

**Affiliations:** Department of Plant Pathology, University of California, Davis, CA 95616 USA

## Abstract

Root-knot nematodes (RKN; *Meloidogyne* spp.) can parasitize over 2,000 plant species and are generally considered to be the most agriculturally damaging group of plant-parasitic nematodes worldwide. Infective juveniles (J2) are non-feeding and must locate and invade a host before their reserves are depleted. However, what attracts J2 to appropriate root entry sites is not known. An aim of this research is to identify semiochemicals that attract RKN to roots. J2 of the three RKN species tested are highly attracted to root tips of both tomato and *Medicago truncatula*. For both hosts, mutants defective in ethylene signaling were found to be more attractive than those of wild type. We determined that cell-free exudates collected from tomato and *M. truncatula* seedling root tips were highly attractive to *M. javanica* J2. Using a pluronic gel-based microassay to monitor chemical fractionation, we determined that for both plant species the active component fractionated similarly and had a mass of ~400 based on size-exclusion chromatography. This characterization is a first step toward identification of a potent and specific attractant from host roots that attracts RKN. Such a compound is potentially a valuable tool for developing novel and safe control strategies.

## Introduction

Root-knot nematodes (*Meloidogyne* spp.; RKN) are sedentary endoparasites that infect many crops and cause substantial losses world-wide^1,2^. Those causing the most damage include the two closely related tropical species, *Meloidogyne incognita* and *M. javanica*, and the temperate-climate species *M. hapla*. Each of these species has a very broad host range, and collectively they parasitize nearly every plant species^2^. RKN infection of crop plants can cause yield losses, collapse of plants, and stunted growth^2^. RKNs suppress host defenses, and, thus, affected plants are more susceptible to other diseases, confounding interpretation of the underlying cause^3^. Measures aimed at controlling plant parasitic nematodes have relied heavily on the use of agrichemicals^4^. Most nematicides are non-specific, notoriously toxic and pose a threat to the soil ecosystem, ground water and human health. The use of fumigant nematicides is becoming increasingly restricted and is steadily decreasing. Host resistance is a desirable alternative but is not available for many crops^5^. Alternative control measures based cultural practices often provide insufficient control. A better understanding of the complex interaction between plant-parasitic nematodes and their hosts is needed to develop new control strategies.

RKN hatch from eggs in the soil into infective juveniles (J2). These J2 are non-feeding and must find a suitable host and establish a feeding site before their reserves are depleted. J2 are preferentially attracted to and penetrate the region of the root just above the root tip, corresponding approximately to the elongation zone^6,7^. After host invasion, they migrate within the root to locate host cells that, in response to signals from the nematode, differentiate into hypertrophied, multinucleated giant cells^8^. However, what attracts nematodes to roots is largely unknown^9,10^. Gradients of physical and chemical factors (e.g., temperature, redox potential, pH and CO_2_) in the rhizosphere influence RKN movement^11,12^, but these factors are not specific to the roots of vascular plants. Under natural conditions, RKN J2 likely respond to multiple sensory inputs perceiving hosts over a long distance through volatile compounds (smell) and more locally through water soluble chemical signals (taste)^13^. There is evidence that plant volatile organic compounds are perceived by *M. incognita* and utilized for host location^14^. While compounds in exudates secreted by roots and/or organisms present in the rhizosphere have been proposed as RKN attractants and repellents^13,15^, the chemical nature of such compounds is largely unknown. Studies of exudates collected from host roots have often found them to be repellent rather than attractive to J2^11,16^, and to date there have been few reports of specific attractants^17,18^.

We have previously utilized a gel of Pluronic F-127 (PF-127) to investigate factors that modulate RKN attraction and behavior^6,12^. PF-127 is a block co-polymer that has low toxicity and forms a thermoreversible gel. A 23% solution of PF-127 is liquid at 15 °C but forms a highly transparent gel at 20 °C. Chemical gradients can be formed in this gel, and nematodes can migrate through the gel and perceive these gradients. Infective juveniles of plant-parasitic nematodes suspended in PF-127 migrate to and accumulate on the roots of host plant seedlings^6^. We also found that J2 of each of the 3 RKN species tested accumulated in a halo surrounding the source of a gradient of acetic acid and that maximum accumulation occurs at pH 4.5–5.4^12^. However, pH gradients are not stable in a soil environment and so likely only contribute to attraction over a very short range.

Using PF-127-based assays, we previously demonstrated that attractiveness of host roots to the temperate-climate RKN species *M. hapla* is modulated by ethylene (ET) signaling^19^. *Arabidopsis* roots impaired in ET synthesis or signaling were more attractive to this species of nematodes, whereas overproduction of ET or constitutive signaling by the plant resulted in reduced attractiveness. Roots of the tomato mutant *Never ripe (Nr)*, which is defective in signaling of the ET response, are also more attractive to *M. hapla* than wild type^19^. However, the effect of the *Nr* mutation on attractiveness of tomato roots to the tropical RKN species *M. incognita* and *M. javanica* was not examined in that study.

The current study was aimed at discovering the plant-emitted compound(s) that guide the J2 to their entry sites just above the root tip. We present evidence that aqueous exudate from seedling root tips is highly attractive to RKN. We also carried out fractionation of tomato and *Medicago* root exudates to characterize the activity and as initial steps toward identification of the active component(s).

## Results

### Attraction of three root-knot nematode species to root tips is affected by ethylene-signaling pathway in the host

**Figure 1:**
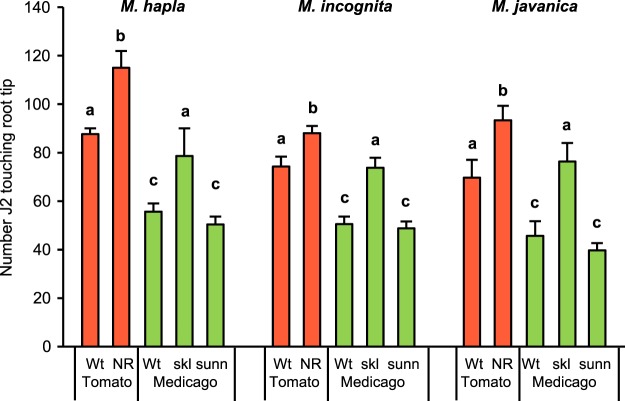
Accumulation of root-knot nematode infective juveniles at root tips. Bars indicate the numbers of infective juveniles (J2) of *Meloidogyne hapla*, *M. incognita*, and *M. javanica* touching the terminal 7 mm of seedling roots after 4 h in 23% PF-127 gel. For each species, the red bars represent the response to wild type (Wt) tomato variety Rutgers and the ethylene insensitive mutant *Never ripe* (NR). The green bars represent attraction to wild type *Medicago truncatula* (Wt) and two hypernodulating mutants, *skl* and *sunn*. Each bar is the mean of 6 replicates with SEM indicated. For each nematode species, different letters above bars represent significantly different numbers of J2 (P < 0.05) according to Tukey’s multiple range test.

In a gel of 23% PF-127, J2 of each of three RKN species, *M. hapla*, *M. incognita*, and *M javanica*, accumulated near root tips of tomato and *M. truncatula* seedlings after a 4 hr exposure (Fig. 1). For each RKN species, more J2 were found touching the terminal 7 mm of roots of the ET-insensitive tomato mutant *Nr* than those of Rutgers, the nearly isogenic wild-type strain. This confirms and extends our previous finding that *Nr* tomato was more attractive to *M. hapla* than Rutgers^19^. Similarly, the hyper-nodulating, ET-insensitive *M. truncatula* mutant *sickle* (*skl*)^20,21^ was more attractive to all three *Meloidogyne* spp than the corresponding wild type genotype. In contrast, the hyper-nodulating mutant *sunn*, which encodes a receptor kinase that is not a component of the ET-signaling pathway^22,23^, did not attract more J2 than the wild type (Fig. 1).

### RKN are attracted to exudates of tomato root tips that were captured in PF-127 gel

**Figure 2:**
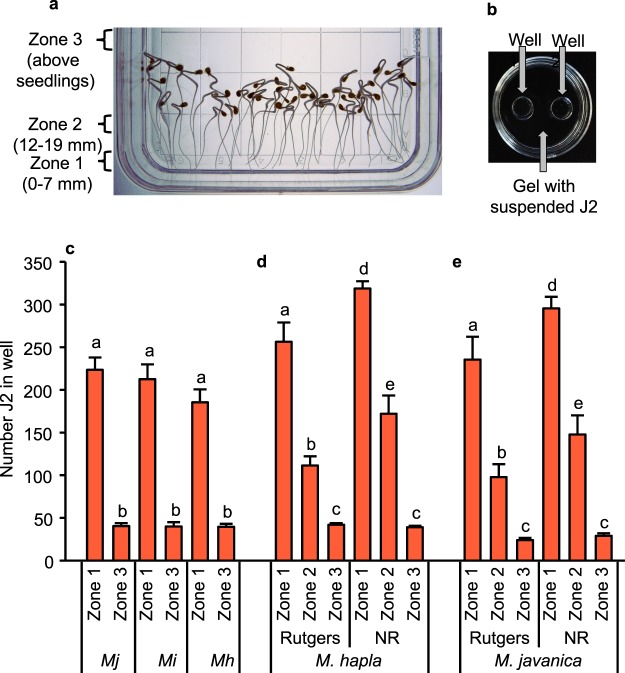
Response of root-knot nematodes to root exudates collected from Pluronic (PF-127) gel. (**a**) Tomato seedlings were aligned in a square, 100 mm Petri plate containing 25 ml of 23% PF-127 gel. Zones are indicated from which gel was collected after 24 hr exposure to seedlings. (**b**) A two well assay was performed in a Petri dish (60 mm). Infective juveniles (J2) were suspended in the gel. Wells were filled with PF-127 gel that had been exposed to root exudate or to controls. The number of juveniles in the wells was counted after 6 hr. Panel (**c**) shows numbers of J2 of *Meloidogyne javanica* (Mj), *M. incognita* (Mi) or *M. hapla* (Mh) accumulating in assay wells to which gel had been transferred from Zone 1 or Zone 3 (n = 6). Panels (**d**) and (**e**) compare accumulation of *M. hapla* and *M. javanica* J2, respectively, in wells receiving gel from Zone 1, Zone 2, and Zone 3 (n = 4). For each graph, letters above bars indicate significantly different numbers of J2 (P < 0.05) according to Tukey’s multiple range test.

We aligned 6 day old tomato seedlings in 23% PF-127 gel (Fig. 2a). After 24 hr, the seedlings were removed, and gel from the area exposed to the terminal 7 mm of roots (Zone 1 in Fig. 2a) and from above the seedlings (Zone 3) was collected. Attraction of nematodes to the extracted gel fractions was assessed using a two well PF-127 gel assay (Fig. 2b). J2 accumulated significantly more in the well containing the gel collected from Zone 1 than in gel from Zone 3, for all three *Meloidogyne* species (Fig. 2c).

In another experiment, we compared the response of *M. javanica* and *M. hapla* to exudates of the ET-insensitive mutant *Nr* and the tomato cv Rutgers. We collected gel from three regions: the terminal 7 mm of root tips (Zone 1), 12 to 19 mm from the root tip (Zone 2) and above the seedlings (Zone 3) (Fig. 2a). For both *M. hapla* and *M. javanica*, PF-127 gel from Zone 1 accumulated more J2 than gel from Zones 2 or 3 (Fig. 2d,e). In addition, PF127 from Zone 1 and Zone 2 of *Nr* tomato accumulated more J2 than the corresponding zones of wild type for both *Meloidogyne* species.

### Characterization of tomato root exudate

**Figure 3:**
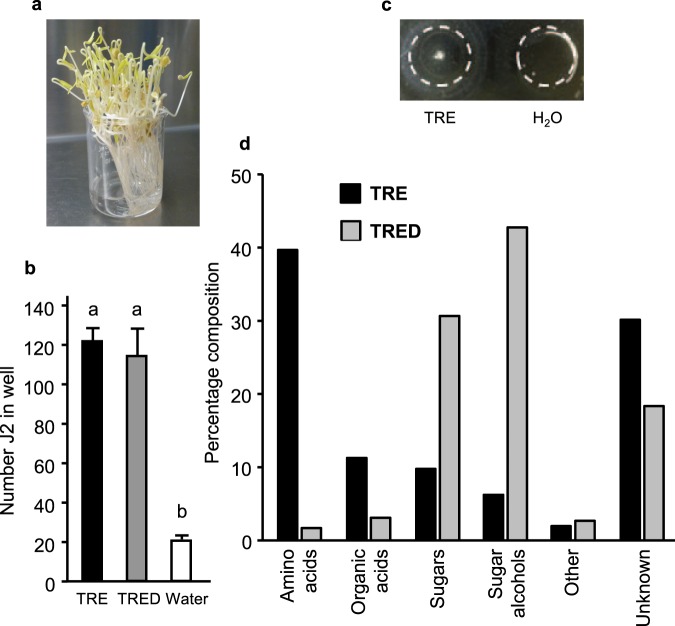
Collection and analysis of tomato root exudate (TRE). (**a**) TRE was collected from root tips in a 20 ml beaker. (**b**) Comparison of accumulation of J2 in TRE and deionized TRE (TRED)-containing wells after 6 h. Letters represent significantly different numbers of J2 (P < 0.05) according to Tukey’s multiple range test. (**c**) J2 aggregated into a clump in TRE-containing gel after 24 h. White dotted lines represent well boundaries. (**d**) Comparison of % composition of major metabolite categories in TRE and TRED.

To obtain PF-127-free material for further analysis, we collected tomato root exudate (TRE) from the root tips of bundled 6 day old seedlings after 24 h in water (Fig. 3a). When PF-127 gel incorporating 20% TRE was assessed in a two well assay, after 6 hr significantly more J2 accumulated in the TRE wells than in the water controls (Fig. 3b). After 24 hr, J2 aggregated into a clump in the TRE-containing well (Fig. 3c). To deplete charged molecules, we passed TRE (pH 4.86) through a mixed bed-resin deionizing column. The deionized TRE was not significantly different from TRE in accumulation of J2 (Fig. 3b) nor was TRE activity significantly diminished following passage through a filter with a molecular mass cut off of 10,000 (not shown).

Primary metabolite analysis of TRE by mass spectrometry detected a complex complement with 193 known and 318 unknown compounds. This analysis reports molecules spanning an approximate molecular weight range of 50 to 600. Amino acids, organic acids, sugars and sugar alcohols were the most abundant categories of known compounds (Fig. 3d). Following passage of the TRE through a mixed bed resin column, amino acids and organic acids were largely depleted. Sugars and sugar alcohols were the most abundant known components remaining. Myo-inositol was about 4.2% of the primary metabolite present in TRE and 10% of that in deionized TRE. Using an enzyme-coupled assay, we determined that the concentration of myo-inositol in TRE was ~70 µM.

### Activity-informed fractionation of tomato and *Medicago* root exudates

**Figure 4:**
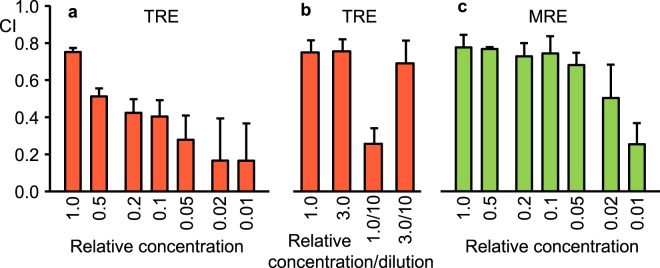
Response of *Meloidogyne javanica* to root exudate concentration. (**a**) Chemotaxis index (CI) for dilutions of tomato root exudate (TRE). (**b**) CI for concentrated and diluted TRE. (**c**) CI for dilutions of *Medicago* root exudate (MRE). A 10 μl microassay was used to obtain the chemotaxis index after 6 h. Each bar represents the average of 3 replicate assays with SD shown.

To monitor the attraction activity during fractionation, we developed a PF-127-based microassay that used 10 µl of test solution per replicate. We used a calculation for chemotaxis index (CI) with a maximum attraction value of 1.0 and a no-response value of 0. Ten µl of a TRE preparation typically gave a CI of 0.7 to 0.8. TRE diluted 20 fold still produced a positive CI (Fig. 4a). Concentrating TRE 3 fold did not increase the CI, indicating that 0.7 to 0.8 was the maximum (plateau) level in this assay. Diluting the 3 fold concentrated TRE 10 fold produced a CI consistent with full recovery of activity (Fig. 4b).

Exudate collected from root tips of *M. truncatula* (*skl*) seedlings was also highly attractive to *M. javanica* J2. Activity of dilutions of *Medicago* root exudate (MRE) revealed a higher activity per root tip than for TRE (Fig. 4c). Using this assay, we found that, as was observed for TRE, MRE was not retained on a mixed bed resin column and passed through a Mr 10,000 cut-off filter. In addition, a C18 reverse-phase resin cartridge did not retain TRE or MRE attraction activity under conditions that retained vitamin B12 and riboflavin. These results suggest that the active components in both TRE and MRE are hydrophilic.

**Figure 5:**
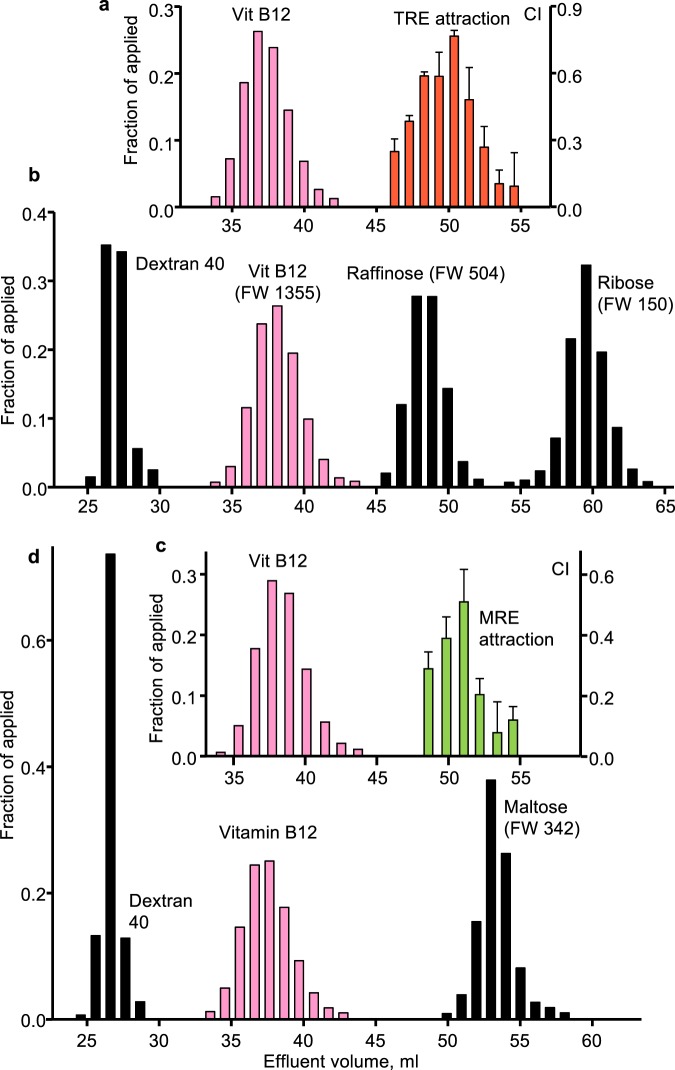
Exclusion chromatography of tomato root exudate (TRE) and *Medicago* root exudate (MRE). The four chromatograms are from separate runs on the same Bio-Gel P2 column. Chromatography was of (**a**) vitamin B12 and TRE, (**b**), dextran 40, vitamin B12, raffinose and ribose, (**c**) vitamin B12 and MRE, and (**d**) dextran 40, vitamin B12 and maltose. Attraction assay results are presented as a chemotaxis index, CI. Results from standards are concentrations expressed as a fraction of the applied concentration. For attraction assays, each bar represents the average of 3 replicate assays with SD shown.

The size exclusion chromatography medium Bio-Gel P-2 is expected to resolve compounds with a molecular weight between 100 and 1800 and has been used to separate oligosaccharides^24,25^. We applied TRE and MRE separately to a Bio-Gel P-2 column that had been calibrated with Dextran 40 (average molecular weight 40,000), vitamin B12 (FW 1355), raffinose (FW 504), maltose (FW 342), and ribose (FW 150) (Fig. 5). Vitamin B12, a convenient internal colored standard found not to attract or repel J2 and to be inert in the attraction assay, was used in all column runs. For both TRE and MRE, the attraction activity eluted as a single peak between raffinose and maltose. The Bio-Gel P-2 column elution profiles of activity in TRE and MRE were indistinguishable, suggesting that the molecular species responsible for the attraction activity in the exudates from these two plant species had a similar or identical mass between 342 and 504.

## Discussion

While previous *in vitro* studies have clearly shown that the root tips of plants are highly attractive to nematodes, identification of the responsible factors has been elusive^13^. We show here that PF-127 gel that has been exposed to tomato root tips is attractive to RKN J2. The ability of PF-127 gel to maintain chemical gradients allowed capture of this activity near the site of exudation from the root. Activity was higher in PF-127 collected from near root tips than from the upper regions of the root. This finding supports the hypothesis that J2 attraction to roots is due to a soluble semiochemical or semiochemicals exuded from the region near root tips and demonstrates that the physical presence of the root tip is not required.

Our work here indicates that attraction of J2 of each of the three RKN species tested is greater to tomato and *Medicago* seedlings that are defective in ET signaling than to the corresponding wild type plants. This supports and extends our previous finding that *M. hapla* J2 were more attracted to ET signaling mutants of *Arabidopsis* and tomato^19^. Interestingly, the soybean cyst nematode, *Heterodera glycines*, is also more attracted to both a non-host (*Arabidopsis*) and host (soybean) compromised in ET signaling^26^. ET regulates diverse plant processes, including responsiveness to stress and pathogen attack^27,28^. The downstream components of the ET-signaling response are part of a complex and highly regulated network involving cross talk with other hormone-signaling pathways^29–31^. The hypernodulating *M. truncatula* mutant *skl* is defective in ET signaling due to a recessive mutation in the gene encoding an ortholog of the Arabidopsis ETHYLENE INSENSITIVE 2 (EIN2) protein, a key positive regulator of ET signaling^21^. We found that this mutant is more attractive than the wildtype to all three *Meloidogyne* species tested. In contrast, another hyper-nodulating mutant *sunn*, which encodes a receptor kinase that is not a component of the ET-signaling pathway^22,23^, did not attract more J2 than the wild type. Compared to the corresponding wild type *M. truncatula* strain A17, roots of *skl* mutants have extensive differences in gene expression and in metabolites produced both constitutively and following pathogen infection^32,33^. Thus, the greater attraction to RKN of *skl* mutants could be due to higher levels of an attractant or lower amounts of repellent components compared to wild type. For example, flavonoids are known to be reduced in *skl* mutants^33^, and these metabolites have been reported to be repellent to RKN J2^34^.

Behaviors and responses of nematodes can be difficult to assess and interpret even for a model system such as *Caenorhabditis elegans*^35^. The complexity of factors involved makes it difficult to design assays and to interpret the response. For example, exudates from root caps of some host plants have been reported to induce quiescence in RKN^36^ rather than to attract or repel the J2. We previously showed that RKN J2 accumulated at pH of ~5, corresponding to the pH at the zone of elongation of actively growing plant roots^12^. This is consistent with the idea that low pH is a local attractant for nematodes. In the current work, we buffered the PF-127 to neutral pH to eliminate this variable. Nematodes also perceive and respond to each other. We have previously observed that, when RKN J2 are suspended in PF-127 gel without roots, they aggregate into tight clumps after 1 to 2 days^37^. The rate of clumping occurs more rapidly at higher nematode density. In our attraction assay, we noted that after 24 h, J2 would frequently form clumps in the well with the attractive exudate fractions (see Fig. 3c, for example). Many nematode species produce pheromones through which they regulate their interactions with each other^38,39^. While RKN J2 release pheromones^40^, their function in nematode behavior, if any, is not yet known. In the assays used in the current paper, nematodes are initially dispersed throughout the gel to minimize J2 interactions.

While some other investigators did not find root exudates to be attractive to RKN J2^11,16^, others did observe attraction^41^. Previous studies generally collected exudate from older roots, which likely had stronger ET signaling, and from the whole root system rather than the tip. Results from our PF-127 experiments suggest that, compared to upper parts of the root, secretions from the root tip area are more attractive. We collected exudate from actively growing root tips of very young seedlings (6 day old for TRE and 2 day old for MRE). For *Medicago*, we use seedlings mutated in ethylene signaling, which may have enhanced the level of attraction observed. However, for tomato, wild type seedlings were used, because insufficient *Nr* seed were available. In the PF-127 assay system, nematodes move through the gel rather than on the surface as they do in the agar plate assays utilized by other investigators^42^. Thus, the PF-127-based assay may favor detection of water soluble attractants rather than volatile components.

Root exudates contain a complex mixture of chemical species including both inorganic and carbon-based compounds of a range of molecular sizes^43^. Primary metabolite analysis of the TRE that we collected revealed more than 500 molecular species, of which fewer than half were identified in reference databases. The composition of the identified components was similar to what has been described for root exudates by others^43^. While we have not yet determined the identity of the attractant(s), we have learned some of its properties. Activity remained following deionization, which resulted in a reduction in amino acids and other ionic compounds and an enrichment for sugars and sugar alcohols. To narrow the size range of candidate compounds, we carried out size exclusion chromatography and found that, for both TRE and MRE, attraction activity eluted as a single peak. Comparison to elution profiles of oligosaccharides suggested that the active component was larger than 342 but smaller than 504. This size range is based on the assumption that the sugar standards and attractant compound(s) have similar relationships of size to hydrodynamic volume and that this component did not interact with the P2 column matrix. An organic molecule in the predicted mass range would not be volatile and would migrate through the soil in water films.

Based on the low concentration of metabolites in the root exudate, the active component appears to be a powerful attractant that can be detected by the nematode at micromolar or lower levels. Since approximately 4 ml of TRE were collected from each bundle of 100 seedlings, 10 µl is predicted to contain the amount of soluble exudate produced by 0.25 seedlings in 24 h, and a 20 fold dilution, which still has detectable activity, would correspond to exudate from 0.0125 seedlings. An even greater dilution of *Medicago* exudate showed attraction. The higher per seedling attraction of MRE compared to TRE could be due to the larger size of the *Medicago* primary root and/or to our use of an ET insensitive mutant line. Nonetheless, the attractive fractions from *Medicago* and tomato have similar properties, suggesting that the same or a similar compound or compounds may attract the J2 to these evolutionarily distant hosts. Identification of the semiochemicals that attract RKN to roots, repel them or otherwise modify their behavior has the potential to provide tools for novel and safe strategies of control. However, identification likely will require scaling up and additional fractionation of seedling root exudates.

## Methods

### Nematode strains and culture

*Meloidogyne incognita* strain VW6, *M. javanica* strain VW4, and *M. hapla* strain VW9 were originally isolated from agricultural locations in California and have been propagated in the greenhouse for over 20 years^37,44,45^. Strains VW4 and VW6 were propagated on tomato (*Solanum lycopersicum* L*.)* cv. *Momor verte*, and *M. hapla* strain VW9 was propagated on tomato cv. *VFNT*. Plants were maintained on separate benches for each species and drip irrigation was used to prevent cross-contamination. Species identification was carried out using morphological and molecular tools and was periodically confirmed by PCR of mitochondrial DNA^46^. In addition, the genome sequence of each of these strains has been determined and supports the species identity^47,48^. Two to three months after inoculation, nematode eggs were collected from roots, and eggs were separated from debris by sucrose flotation and allowed to hatch in a hatching chamber as described^49^. J2 hatched after 48 h were rinsed three times with sterile water using a Millipore Steriflip™ Disposable Vacuum Filter unit (Millipore Corporation, Bedford, USA). The J2 retained on the filter were collected and then used immediately in the assays.

### Plant material

Tomato seeds of *Rutgers*, *Never ripe* (Rutgers background) and *Moneymaker* were gifts from R. M. Bostock and from K. Bradford, both of the University of California, Davis, USA. Seeds of *Medicago truncatula* cv Jemalong A17 (wild-type), and the mutants *sunn* and *sickle* (*skl)*^20,22^ were provided by D. Cook, University of California, Davis. Tomato seeds were surface-sterilized in 2:1 ethanol:water solution for 3 min followed by treatment with 4% sodium hypochlorite for 10 min and rinsed in water three times for 5 min each^19^. For germination, tomato seeds were spread on moist, sterile Whatman No. I filter paper disks in Petri dishes and incubated in the dark at 25 °C. *Medicago* seeds were scarified mechanically or by acid treatment and then surface sterilized with 0.6% sodium hypochlorite and germinated on 0.8% agar^50^.

### Root attraction assays

Attraction to seedling root tips was assessed as described^6^. Freshly hatched RKN J2 were suspended at approximately 200 per ml in 23% (wt/vol) PF-127 (Sigma-Aldrich, USA) augmented with a buffer composed of equimolar Tris and morpholinoethanesulfonic acid (MES) (one mM each or as indicated in text), unadusted pH7.1 (TM7). Five ml of suspension were delivered to each well of a 6 well tissue culture plate (#353046, Corning Inc., Corning, NY, USA) at 15 °C. One seedling (5 day old for tomato or 3 day old for *Medicago*) was introduced in the center of each well, and the plates were transferred to room temperature. After 4 h, the number of J2 touching the terminal 7 mm of the root was counted. Each treatment was replicated six times, and the experiments repeated at least four times.

### Collection of root-exposed Pluronic gel

Fifty 6 day old tomato seedlings of each cultivar tested were lined up in a 100 × 100 mm Petri plate containing 25 ml of 23% PF-127 solution, 1 mM TM7, at 15 °C. The PF-127 was allowed to gel at room temperature. The Petri plate was sealed with Parafilm® paraffin sheet and incubated in the dark at 25 °C for 24 h after which the seedlings were removed. The gel from the indicated regions was collected with a spatula, liquified by chilling and passed through a 0.2 µm cellulose acetate membrane syringe filter (Millipore Corporation, Bedford, USA) to remove cells.

### Collection and analysis of tomato root exudate (TRE) and Medicago root extract (MRE)

The root tips of 100 six day old tomato seedlings (cv. *Moneymaker*) were aligned on moistened strips (2 cm by 12 cm) of autoclaved Ultra Clear cellophane (Research Products International Corp.) and covered with another cellophane strip. The seedlings were then rolled together such that the terminal 10 mm of the roots were exposed. The bundled seedlings were suspended in a 20 ml beaker with 4 ml of sterile water to submerge the root tips (Fig. 3a). Each 20 ml beaker was then placed in a 250 ml beaker with a wet Kimwipe® tissue at the bottom and the whole setup was covered by sheet paraffin with a few pin holes for ventilation. After 24 h at 25 °C, the liquid in the beakers was collected and passed through a 0.2 µm filter. This TRE was stored at −20 or −80 °C. Another 4 ml of water was added to the 10 ml beaker and a second collection of TRE was make after another 24 h. The two collections showed similar attraction activity. MRE was collected similarly except that the seedlings were 2 days old, and incubation was at 20 °C.

Where indicated, exudate was filtered through a 10,000 NMWC filter (Micron Separations INC., Westboro, MA, USA). Myo-inositol concentration was determined using an enzyme-based assay kit (K-INOSL, Megazyme, Bray, Ireland). Where indicated, root exudate was concentrated in Eppendorf tubes that were centrifuged in a Speed Vac Concentrator (Savant) while being exposed to a vacuum of 100–500 mTorr.

### Two well assay for exudate activity

For each assay, 10 ml of 23% PF-127 solution 10 mM TM7 was mixed thoroughly with 3000 freshly hatched J2 at 15 °C. This nematode suspension was added to a 60 mm diameter Petri plate containing 2 vertically placed glass cylinders (10 mm in diameter and 20 mm apart) at room temperature. After the gel solidified, the cylinders were removed leaving two wells. Wells were filled with 0.35 ml of 23% PF-127 containing 10 mM TM7 and control or test solution. The assay was incubated at 25 °C in the dark and the number of J2 within each well was counted under a dissecting microscope at indicated times after the wells were filled.

### Micro assay for exudate activity

For each well of a 6 well tissue culture plate, 3 ml of 23% Pluronic F-127 solution in 10 mM TM7 was mixed thoroughly with 1500 freshly hatched J2 at 15 °C. After the gel solidified at room temperature, 10 µl of test solution and 10 µl water or other control as indicated were injected into the gel at marked points 1.5 cm apart. Plates were incubated at 25 °C in the dark. The number of J2 within a 5 mm circle centered on the injection site of the test solution or control was counted under a dissecting microscope after 6 h. Chemotactic index (CI) was presented as (A − B)/(A + B) where A is the number of J2 counted in a 5 mm circle centered on the point of injection of the test substance and B is the number in the control area. Three replicate assays were used for each data point.

### Deionization of TRE

Aqueous fractions were passed through a 0.5 ml bed volume, 7 mm inside diameter column containing Mixed Bed Resin (M8032, Sigma-Aldrich). Zero point eight ml of TRE were applied to the top of the resin bed and the column was centrifuged at 3000 rpm (1000×*g*) for 1–2 min to collect the filtrate. Using the same column, this step was repeated with a second 0.8 ml aliquot of TRE.

### GC-MS analysis of TRE

Gas Chromatography TOF Mass Spectrometry analysis (GC-MS) for primary metabolites was carried out by Genome Center Core Services at the University of California, Davis. Samples were dried and subjected to methoximation and trimethylsilylation derivatization as described^51^. All samples were spiked with a mixture of fatty acid methyl esters as internal standards^51,52^. An Agilent 6890 gas chromatograph (Santa Clara, California, USA) containing a 30 m long, 0.25 mm inside diameter Rtx-5Sil MS column (Restek Corporation, USA) with an additional 10 m integrated guard column was used to run the samples. Absolute spectra intensities were processed by a filtering algorithm implemented in the metabolomics BinBase database^53^. Quantification, metabolite assignment and data normalization were carried out as described^52^. Metabolites were placed into categories (amino acids, organic acids, sugars, sugar alcohols, other and unknown), and percentage composition was estimated from peak height compared to total metabolite peak height. “Unknowns” refer to peaks that could not be assigned in available metabolite libraries.

### Reverse phase chromatography

Aqueous exudate spiked with (0.1 mM riboflavin or vitamin B12) as a control for binding was applied to a Sep-Pak ® Plus C18 cartridge (WAT020515, Waters, Milford, MA, USA) following conditioning as recommended by manufacturer. Fractions were collected following sequential washes with 10 mM TM7, water, then 20, 50, and 100% methanol.

### Gel exclusion chromatography

Bio-Gel P2 (BioRad, USA) polyacrylamide gel beads were swelled and poured into a glass chromatography column according to the manufacturer’s suggested practices. The column (14 mm inside diameter and 77 ml bed volume) was equilibrated with at least two bed volumes of 10 mM TM7 and eluted with the same buffer. Column was operated at a linear flow rate of between 5 and 5.5 cm/h under gravity flow. Standards were incorporated at the following concentrations into the 0.8 ml sample that was applied to the column: dextran 40, 1 mg/ml; vitamin B12, 2 A_360_; raffinose, 4 mM; maltose, 10 mM; ribose, 7.5 mM. For column runs with carbohydrate standards, aliquots of the fractions eluted from the column were assayed for carbohydrate content using a sulfuric acid and phenol method as described^54^ except that 30 µl of 5% phenol were added to the 50 µl sample in a 96 well plate before, rather than after, the addition of 150 µl of concentrated sulfuric acid. Values were compared to polysaccharide and sugar calibration standards.

### Statistical analysis

Two factor ANOVA was carried out with R Statistical Software (version 3.1.3; R Foundation for Statistical Computing, Vienna, Austria). Tukey’s multiple range test was conducted to identify significant differences (P < 0.05) between the treatment means.
